# Influence of Probiotics on the Development of *Clostridioides difficile* Infection in Patients Receiving Fluoroquinolones

**DOI:** 10.3390/pharmacy9030141

**Published:** 2021-08-18

**Authors:** Mary E. Sheffield, Bruce M. Jones, Blake Terrell, Jamie L. Wagner, Christopher M. Bland

**Affiliations:** 1 St. Joseph’s/Candler Health System, Savannah, GA 31405, USA; cmbland@uga.edu; 2 Department of Clinical and Administrative Pharmacy, University of Georgia College of Pharmacy, Savannah, GA 31324, USA; blake.terrell25@uga.edu; 3 Department of Pharmacy Practice, University of Mississippi School of Pharmacy, Jackson, MS 39216, USA; jwagner@umc.edu

**Keywords:** antibiotic-associated diarrhea, CDI, ciprofloxacin, *Clostridioides difficile*, levofloxacin, probiotics

## Abstract

Fluoroquinolones are associated with an increased risk of *Clostridioides difficile* infection (CDI). Probiotic supplementation has been shown to reduce the risk of antibiotic-associated diarrhea with variable effects on CDI. The objective of this study was to evaluate receipt of probiotics on development of primary CDI among hospitalized patients receiving fluoroquinolones. A retrospective cohort was evaluated that consisted of two groups of 100 patients each, admitted August 2018 through August 2020 that received ≥3 days of definitive monotherapy with levofloxacin or ciprofloxacin within 72 h of admission. Primary outcome was incidence of CDI. Secondary outcomes included rates of *C. difficile* diagnostic stool testing, additional infectious diagnostic testing, and non-CDI related gastrointestinal side effects. Patients on fluoroquinolones who received probiotics had a non-statistically significantly lower incidence in overall cases of CDI compared to those who did not receive probiotics (0% vs. 3%, *p* = 0.246). Patients who received probiotics had statistically significantly fewer *C. difficile* diagnostic stool tests performed (4% vs. 16%, *p* = 0.005) and fewer additional infectious diagnostic testing performed (4% vs. 10%, *p* = 0.096), respectively. Further research is warranted to optimize and standardize probiotic prescribing in high-risk patients.

## 1. Introduction

*Clostridioides difficile*, previously *Clostridium difficile*, is a Gram-positive, anaerobic, spore-forming bacterium responsible for *C. difficile* infection (CDI), including the development of pseudomembranous colitis and toxic megacolon [1]. Complications include severe diarrhea, dehydration, sepsis, and even death. In 2014, CDI became recognized as the leading cause of hospital-acquired infections in the United States [2]. Primary prevention of CDI is of interest due to the risk of recurrence (~20–25%) and associated health consequences, including increased morbidity and mortality, hospital length of stay, and healthcare costs [3].

The primary risk factor for development of CDI is antibiotic exposure. Risk varies based on antibiotic classes and patient risk factors; however, the highest associated risk has been identified with the use of clindamycin, fluoroquinolones, and ceftriaxone [1,4]. Fluoroquinolones are one of the most frequently and inappropriately prescribed antibiotic classes in the United States. Recent data published by the CDC revealed that despite antimicrobial stewardship efforts, 47% of inpatient fluoroquinolone use is inappropriate [5]. Additionally, fluoroquinolone use has been associated with increased prevalence of the *C. difficile* ribotype 027 strain, which has significantly higher rates of morbidity and mortality [1].

Probiotic supplementation has been shown to reduce the risk of antibiotic-associated diarrhea and variable effects on primary CDI [6,7,8,9,10]. Due to conflicting and insufficient data, often due to poor quality studies, routine use is not recommended per the 2018 Infectious Diseases Society of America (IDSA) guidelines [11]. The biggest potential impact for use in clinical practice could be among patients receiving high-risk antibiotics, but data are limited. The aim of this study was to evaluate the receipt of probiotics on the development of primary CDI among patients receiving fluoroquinolones compared to those who did not receive probiotics.

## 2. Materials and Methods

### 2.1. Setting and Study Design

This multi-center, retrospective, observational cohort was conducted after institutional review board approval from St. Joseph’s/Candler Health System, a 714-bed not-for-profit, comprehensive, community health system consisting of two hospitals. A computer-generated list identified admitted patients who received intravenous (IV) or oral levofloxacin or ciprofloxacin from 1 August 2018 to 31 August 2020. Ciprofloxacin and levofloxacin were specifically evaluated from the fluoroquinolone class as they are the main two formulary agents used within the health system. Included patients were randomized into two groups of 100 patients, each based on concomitant use of at least one dose of probiotic(s) during definitive therapy, versus those that did not receive probiotics. The two probiotics used during the study period were *Saccharomyces boulardii* and a *Lactobacillus* spp. predominant blend of *Lactobacillus acidophilus*, *Lactobacillus bulgaricus*, *Bifidobacterium bifidum*, and *Streptococcus thermophiles* (Risa-Bid^®^)(Rising Pharmaceuticals, Allendale, NJ, USA). Probiotic selection varied based on product availability and provider preference. Dosing regimens were not standardized and were up to the discretion of the treating physician.

Patients were included if they were ≥18 years of age and received at least 3 days of definitive monotherapy with IV or oral levofloxacin or ciprofloxacin within 72 h of hospital admission. Patients were excluded if they had a documented history of prior CDI, antibiotic use in the outpatient setting within 90 days of hospitalization, co-administration of additional systemic antibiotics for more than 24 h during definitive therapy, immunocompromised, history of inflammatory bowel disease or irritable bowel syndrome, or pregnancy. The primary outcome was the incidence of primary CDI, defined as symptomatic patients with positive stool testing. Symptomatic was defined as three or more episodes of diarrhea with an elevated white blood cell count (WBC) or fever, 7 or more bowel movements, or 1.5 L of stool output over a 24-h period [11,12]. Secondary outcomes evaluated were rates of *C. difficile* diagnostic stool testing performed, rates of additional infectious diagnostic testing performed, and rates of non-*C. difficile* related gastrointestinal (GI) side effects.

Within this institution, *C. difficile* stool testing can be ordered directly by physicians, or via a nurse-driven protocol under which nurses may order testing within the first three days of admission. The protocol requires three or more episodes of diarrhea in a 24 h period and one additional symptom of CDI (WBC ≥ 15,000 K/mm^3^, temperature ≥ 38 °C, loss of appetite, nausea, or abdominal pain or tenderness). Stool samples are processed with a 2-step algorithm. A combined glutamate dehydrogenase (GDH)/Toxin A/B test is completed first. A reflex polymerase chain reaction (PCR) test is performed if the GDH is positive, but the toxin is negative.

### 2.2. Demographics and Patient Characteristics

Patient demographic data included age, sex, and race. Charlson Comorbidity Index scores were calculated to compare confounding risk factors between both groups. Objective laboratory values collected included WBC, C-reactive protein, erythrocyte sedimentation rate, serum creatinine, procalcitonin, and albumin. Data regarding antibiotic and probiotic administration, including product used, timing of initial doses, number of doses administered, duration of therapy, and use of proton pump inhibitors (PPI) or histamine-2 receptor antagonists (H2RA) was also obtained. Additional infectious diagnostic testing included GI PCR panel, stool culture, fecal fat test, fecal occult blood test, stool WBCs, or repeat imaging.

### 2.3. Statistical Analysis

Data was collected in REDCap^®^ (version 9.6.3, Vanderbilt University, Nashville, TN, USA). Bivariate data were evaluated using chi-square test and Fisher’s exact test for nominal data, as appropriate. Continuous data were evaluated using Mann–Whitney *U* test. A two-sided alpha value of 0.05 was deemed statistically significant. All data were analyzed using SPSS version 27.0 (IBM).

## 3. Results

There were 1104 patients who received IV or oral ciprofloxacin or levofloxacin who were screened for inclusion to obtain 100 eligible patients in each group. Of the 1104 screened, 904 patients were excluded based on less than 3 days of IV or oral ciprofloxacin or levofloxacin (46%), additional systemic antibiotics for more than 24 h during definitive therapy (32%), and antibiotic use in the outpatient setting within 90 days of hospitalization (9%). Patient demographics and clinical characteristics are summarized in Table 1. Between both groups, the median patient age was 67 years, 62% were female, and the median Charlson Comorbidity Index score was four. Use of levofloxacin versus ciprofloxacin was consistent across both groups with a mean duration of seven days.

Higher percentage of PPI use in the non-probiotic group was observed (61% vs. 41%, *p* = 0.005). Antibiotic use prior to switching to definitive therapy was 51% in the probiotics group and 9% in the non-probiotic group (*p* < 0.001). Probiotic dosing or frequency has not yet been standardized at our institution. However, most patients who received probiotics were already receiving a daily probiotic or started probiotics at the start of fluoroquinolone therapy. Of the patients receiving probiotics, 76% received Lactobacillus spp., 22% received S. boulardii, and 2% received doses of both probiotics.

**Figure 1:**
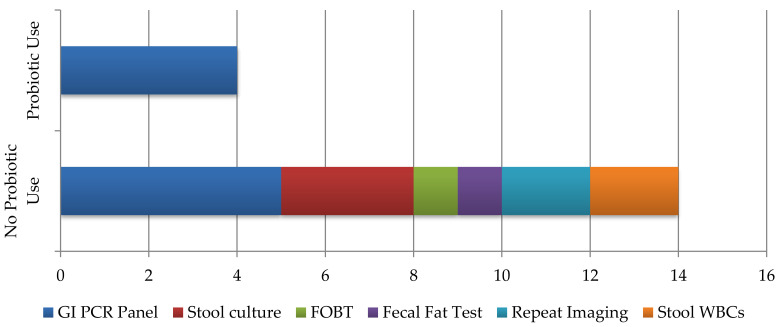
Rates of additional infectious diagnostic testing ordered. Abbreviations: GI, gastrointestinal; PCR, polymerase chain reaction; FOBT, fecal occult blood test; WBC, white blood cell.

For the primary outcome, patients on fluoroquinolones who received probiotics had a non-statistically significant lower incidence in overall cases of CDI compared to those who did not receive probiotics (0% vs. 3%, *p* = 0.246). Regarding secondary outcomes, patients who received probiotics had statistically significantly fewer C. difficile diagnostic stool tests performed compared to those who did not receive probiotics (4% vs. 16%, *p* = 0.005). Additionally, patients receiving probiotics had fewer additional infectious diagnostic testing performed compared to those who did not receive probiotics (4% vs. 10%, *p* = 0.096) (Figure 1). Between both groups, approximately 70% of patients reported no gastrointestinal-related side effects during their admission. Of the 30% and 35% of patients who did experience side effects, vomiting was statistically significantly higher in the non-probiotic group (9% vs. 2%, *p* = 0.030) (Table 2).

## 4. Discussion

Overall, this study provides further insight into the use of probiotics for primary prevention of CDI in high-risk patients, for which there are currently limited data. Although not statistically significant, zero cases of CDI were observed among patients on fluoroquinolones who received probiotics. The rate of CDI observed in patients receiving fluoroquinolones without probiotics is consistent with the current literature. The reported incidence of hospital-onset CDI has been shown to be approximately 8 per 10,000 patient-days [13,14]. Great interest surrounds the potential use of probiotics for prevention of CDI. Not only is CDI associated with a significant increase in healthcare cost and hospital length of stay, it is also a Center for Medicare and Medicaid Service’s (CMS) core measure. In 2016, healthcare facility-onset CDI was added to the CMS hospital-acquired condition reduction program. Hospitals that do not meet the 75-percentile cut off are subjected to CMS reimbursement penalties, furthering the financial burden on these institutions [15].

The use of probiotics has been shown to have beneficial effects on the GI tract through various proposed mechanisms, including restricting pathogenic growth by competing for essential nutrients, inhibiting adhesion of *C. difficile* in the intestine, producing antimicrobial metabolites, reducing osmotic diarrhea and restoring intestinal metabolic homeostasis [16,17]. Hudson et al. retrospectively evaluated prophylactic probiotic use in patients receiving broad-spectrum antibiotics for at least 3 days. In the 2.5-year study period, 5574 hospital encounters were reviewed and showed a *C. difficile*-associated diarrhea (CDAD) incidence rate of 0.96% in patients who received probiotics compared to 2.19% in patients who did not receive probiotics (*p* = 0.007; number needed to treat of 88) [6]. A 2018 meta-analysis and systematic review by McFarland et al. evaluated three randomized controlled trials and seven observational studies, and showed a reduced incidence rate of CDI with use of Bio-K+^®^ for primary prevention of CDI [7]. Shen et al. analyzed data from 19 studies and concluded that the initiation of probiotics within two days of antibiotics reduced the risk of CDI by greater than 50%. A greater risk reduction was evident when probiotics were given within two days of antibiotic initiation compared to 3–7 days (*p* = 0.02) [8]. Johnson et al. evaluated 11 studies and overall showed a protective effect against CDI (RR 0.39), especially with administration of Bio-K+^®^ probiotics compared to placebo (RR 0.21) [9]. Goldenberg et al. conducted a meta-analysis of 31 randomized controlled trials and concluded that probiotics significantly reduce CDI risk compared to placebo with a number needed to treat of 12. Post hoc subgroup analyses showed probiotics were only effective among trials with a > 5% baseline CDI risk [10].

These positive results were directly challenged with the publication of the PLACIDE trial in 2013, the largest randomized control trial for this population to date. Allen et al. failed to show a statistically significant benefit of probiotics in the prevention of antibiotic-associated diarrhea (AAD) or CDI [18]. A recent multi-center trial by Heil et al. evaluated the impact of a computerized clinical decision support tool to prescribe probiotics to high-risk patients for primary prevention of CDI [19]. Over the 13-month post intervention period, 2489 patients (16.6%) received probiotics and no difference in CDI was observed compared to the pre-intervention period. A propensity-score match evaluation was performed and patients who received probiotics did not have lower rates of CDI compared to patients who did not receive probiotics (RR 1.46; 95% CI 0.87-2.45; *p* = 0.15). These results from Heil et al. further demonstrate a lack of impact of probiotics on CDI primary prevention. Variations in study designs, probiotic formulations, duration of therapy, baseline rates of CDI, and inclusion of low-risk patient populations, provide additional challenges when directly comparing between studies. To our knowledge, this is the first study to examine the use of probiotics with fluoroquinolones specifically. The unique design of the trial allowed us to investigate the impact of probiotics within a specific high-risk patient population that has not been previously studied. With widespread use of fluoroquinolones, we believe these results could potentially translate to other health systems.

Within our secondary outcomes, we did identify a statistically and clinically significant decrease in *C. difficile* diagnostic stool testing as well as additional infectious diagnostic testing. Reported symptoms overall trended down with probiotic use, with a statistically significant decrease in vomiting. Together these secondary results imply that probiotic use may reduce frequency and severity of AAD and GI side effects to a tolerable level, avoiding additional and un-necessary patient work up. Utilization of nursing protocols featuring broad, generalized symptoms like nausea and abdominal pain for CDI testing criteria likely attribute to excess testing. While probiotics may or may not reduce the incidence of primary CDI, there could be a potential pharmacoeconomic benefit by avoiding adverse drug reactions and conserving hospital resources. These findings introduce a new and meaningful outcome worth evaluating in CDI trials going forward.

Probiotic dosing and frequency have not yet been standardized at our institution. Most patients who received probiotics were already receiving a daily probiotic as continuation of a home medication or started probiotics at the start of fluoroquinolone therapy based on provider discretion. Standardization of probiotic use is of interest among many hospital institutions due to the potential cost savings benefit and improved CMS measure compliance. However, the literature provides little guidance for standardization of use, patient selection, or product choice. Additionally, implementation of new protocols may prove to be difficult without more robust data. As shown in Heil et al., only 16.6% of eligible patients received probiotics over the 13-month intervention period.

Several limitations are present within this study. The retrospective nature of this study design limited the ability to control confounding factors amongst non-standardized use of probiotics from prescribing physicians. There was a higher percentage of PPI use in the non-probiotic group, which is a risk factor for CDI. However, patients in the probiotic group had a much higher percentage of antibiotic use prior to switching to definitive therapy. These confounding factors may have influenced the findings of this study. The inclusion and exclusion criteria were chosen to focus the aim of this study on a specific population with high risk and frequent use.

## 5. Conclusions

Although not statistically significant, zero cases of CDI were observed among patients on fluoroquinolones who received probiotics. The role of probiotics for primary prevention of CDI remains unclear. When administered to patients receiving fluoroquinolones, the use of probiotics resulted in a statistically and clinically significant decrease in diagnostic stool testing, without an increase in side effects. With the continued inappropriate and excessive use of fluoroquinolones, the results of this trial provide important data in a tangible target population. Further research is warranted to optimize and standardize probiotic use specifically in high-risk patients.

## Figures and Tables

**Table 1:** Demographics and patient characteristics. ^†^ Median (interquartile range) unless otherwise noted. * Two patients received doses of both probiotics. Abbreviations: PPI, proton pump inhibitor; H2RA, histamine-2 receptor antagonist; BID; twice daily; TID, three times a day; FQ, fluoroquinolone.

Characteristic ^†^	Probiotic Use (*n* = 100)	No Probiotic Use (*n* = 100)	*p*-Value
Age, years	68 (57–78)	64 (55–74.75)	0.120
Male, no. (%)	41 (41)	35 (35)	0.382
Race, no. (%)			
Caucasian	72 (72)	57 (57)	0.027
Black	28 (28)	41 (41)	0.053
Hispanic	0 (0)	1 (1)	1.000
American Indian/Alaskan Native	0 (0)	1 (1)	1.000
Charlson comorbidity index	4 (2–5)	4 (2–5)	0.652
Definitive monotherapy, no. (%)			0.670
Levofloxacin	53 (53)	56 (56)	
Ciprofloxacin	47 (47)	44 (44)	
Fluoroquinolone duration, days	7 (5–10)	7 (5–9)	0.277
PPI use, no. (%)	41 (41)	61 (61)	0.005
H2RA use, no. (%)	26 (26)	25 (25)	0.871
Prior antibiotic use, no. (%)	51 (51)	9 (9)	<0.001
Probiotic use, no. (%) *			
*Lactobacillus*	76 (76)	-	
*Saccharomyces*	22 (22)	-	
Probiotic frequency, no. (%)			
Daily	30 (30)	-	
BID	24 (24)	-	
TID	44 (44)	-	
Other	2 (2)	-	
Duration of probiotics, days	6 (4–9)	-	
Time from the start of FQ to first probiotic dose, days	0 (0,1)	-	

**Table 2:** Incidence of non-*C. difficile* related gastrointestinal side effects. ^†^ Abdominal pain (*n* = 1), constipation (*n* = 1).

Symptoms	Probiotic Use (*n* = 100)	No Probiotic Use (*n* = 100)	*p*-Value
No Symptoms	70 (70)	65 (65)	0.450
Nausea	11 (11)	13 (13)	0.663
Vomiting	2 (2)	9 (9)	0.030
Bloating	3 (3)	4 (4)	1.000
Gas	6 (6)	10 (10)	0.297
Non-CDI diarrhea	17 (17)	20 (20)	0.585
Other	0 (0)	2 (2) ^†^	0.497

## Data Availability

The data presented in this study are available upon request from the corresponding authors.
